# Severe mitral regurgitation with bidirectional Coanda effect

**DOI:** 10.1007/s12471-026-02034-w

**Published:** 2026-03-16

**Authors:** Boudewijn Klop, Naomi M. A. J. Timmermans, A. Ramon T. van de Ven, Niels Verberkmoes, Stijn de Ridder

**Affiliations:** 1 Department of Cardiology, Anna Hospital, Geldrop, The Netherlands; 2 Department of Cardio-Thoracic Surgery, Catharina Hospital, Eindhoven, The Netherlands

## Answer

**Fig. 1:**
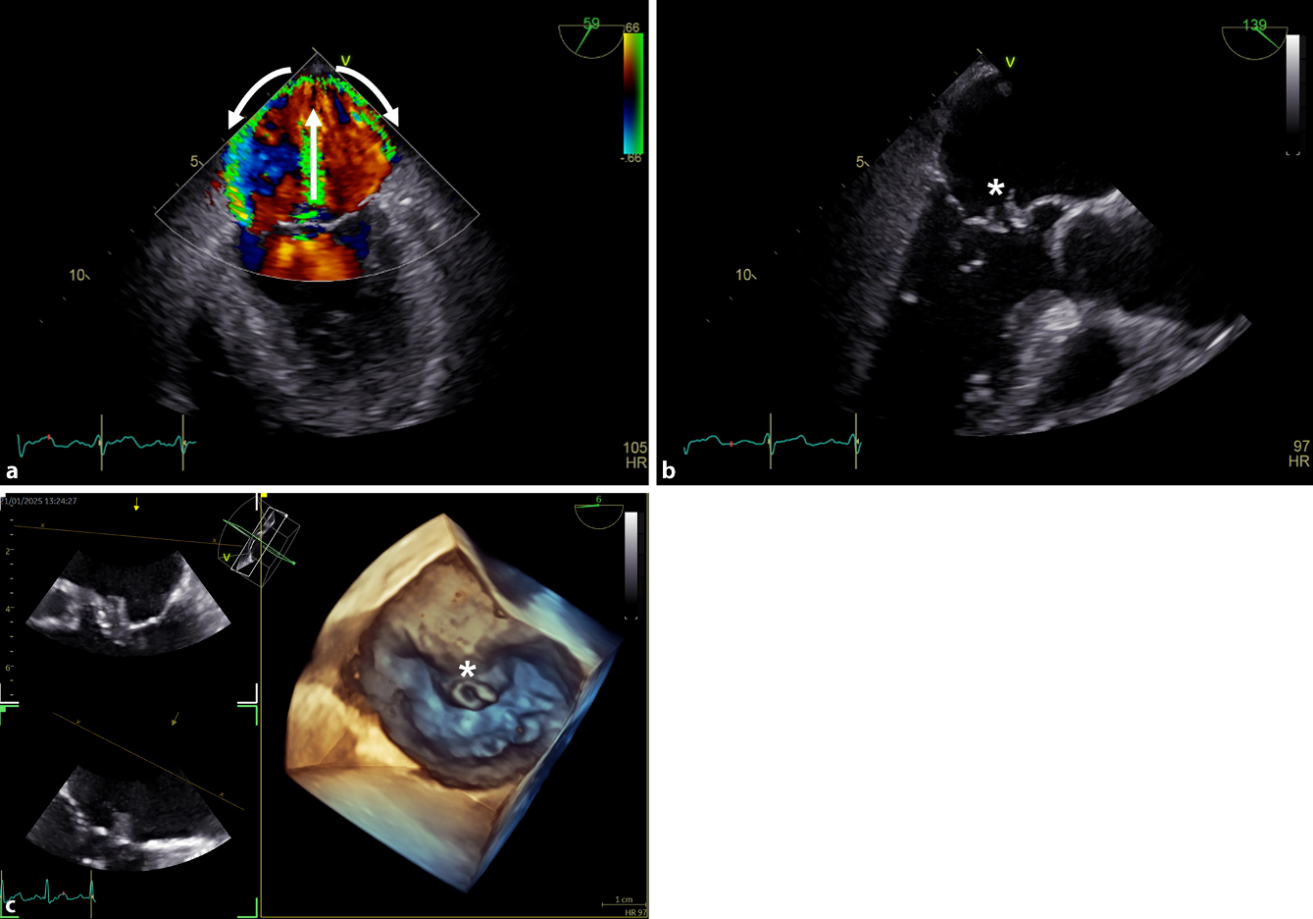
The arrows point out the direction of the mitral valve regurgitation with laminar flow and its symmetric, bidirectional Coanda effect in the mid-esophageal commissural view (**a**). Two-dimensional mid-esophageal aortic valve long-axis view (**b**) and three-dimensional surgical view of the mitral valve (**c**) showing the vegetation and tunnellike perforation of the anterior mitral valve leaflet (*)

The trans-esophageal echocardiogram showed a severe mitral regurgitation due to a tunnellike perforation of the anterior valve leaflet at the site of a large vegetation (Fig. 1). The severe aortic regurgitation and abscess of the sinus of Valsalva remained unchanged. The movement of the mitral valve leaflets was normal. Therefore, the mitral regurgitation should be classified as type 1 according to the Carpentier classification [1]. Color Doppler showed a mitral regurgitation with a symmetric, bidirectional Coanda effect, which advanced from the posterior wall along both the medial and lateral walls of the left atrium back towards the mitral annulus. A Coanda effect is regularly observed with excentric mitral regurgitation, where the regurgitant blood follows the adjacent curved surface of the left atrium. This phenomenon was named after the Romanian inventor Henri Coandă [2]. However, the presence of a symmetric, bidirectional Coanda effect in our case is extremely rare and has not been described in medical literature before, to our knowledge. The mechanism of the symmetrical, bidirectional Coanda effect of the mitral regurgitation was probably due to its severity and unidirectional flow through the tunnellike perforation with perfect alignment of the regurgitation against the posterior wall of the left atrium at a 90 degree angle, which then spreaded out over the medial and left lateral wall (Fig. 1 and online video supplements B, C and D).

The patient was successfully treated with an urgent mitral valve repair and bioprosthetic aortic valve replacement together with antibiotic treatment using intravenous benzylpenicillin up to two weeks after surgery.

## Supplementary Information

**Online video supplement B**: Two-dimensional mid-esophageal commissural view of the mitral valve with color Doppler.

**Online video supplement C**: Two-dimensional mid-esophageal aortic valve long-axis view showing the vegetation and tunnellike perforation of the anterior mitral valve leaflet.

**Online video supplement D**: Three-dimensional surgical view of the mitral valve showing the vegetation and tunnellike perforation of the anterior mitral valve leaflet.
